# Evaluation of FRESH scores in predicting outcome and quality of life after aneurysmal subarachnoid haemorrhage in a European patient cohort

**DOI:** 10.1007/s00701-024-05909-2

**Published:** 2024-01-23

**Authors:** Björn B. Hofmann, Evgenia P. Gundlach, Igor Fischer, Sajjad Muhammad, Rainer Kram, Kerim Beseoglu, Jan F. Cornelius

**Affiliations:** 1 Department of Neurosurgery, Medical Faculty and University Hospital Düsseldorf, Heinrich-Heine-University, Düsseldorf, Germany; 2 Department of Anesthesiology, Medical Faculty and University Hospital Düsseldorf, Heinrich-Heine-University, Düsseldorf, Germany

**Keywords:** Aneurysmal subarachnoid haemorrhage, Patient-reported outcome, Quality of life (QoL), SF-36

## Abstract

**Background:**

Despite aneurysmal subarachnoid haemorrhage (aSAH) patients often experiencing physical and mental disabilities impacting their quality of life (QoL), routine assessment of long-term QoL data and predictive tools are limited. This study evaluates the newly developed “functional recovery expected after subarachnoid haemorrhage” (FRESH) scores with long-term outcomes and QoL in European aSAH patients.

**Methods:**

FRESH, FRESH-cog, and FRESH-quol scores were retrospectively obtained from aSAH patients. Patients were contacted, and the modified Rankin Scale (mRS), extended short form-36 (SF-36), and telephone interview for cognitive status (TICS) were collected and performed. The prognostic and empirical outcomes were compared.

**Results:**

Out of 374 patients, 171 patients (54.1%) completed the SF-36, and 154 patients completed the TICS. The SF-36 analysis showed that 32.7% had below-average physical component summary (PCS) scores, and 39.8% had below-average mental component summary (MCS) scores. There was no significant correlation between the FRESH score and PCS (*p* = 0.09736), MCS (*p* = 0.1796), TICS (*p* = 0.7484), or mRS 10–82 months (average 46 months) post bleeding (*p* = 0.024), respectively. There was also no significant correlation found for “FRESH-cog vs. TICS” (*p* = 0.0311), “FRESH-quol vs. PCS” (*p* = 0.0204), “FRESH-quol vs. MCS” (*p* = 0.1361) and “FRESH-quol vs. TICS” (*p* = 0.1608).

**Conclusions:**

This study found no correlation between FRESH scores and validated QoL tools in a European population of aSAH patients. The study highlights the complexity of reliable long-term QoL prognostication in aSAH patients and emphasises the need for further prospective research to also focus on QoL as an important outcome parameter.

**Supplementary Information:**

The online version contains supplementary material available at 10.1007/s00701-024-05909-2.

## Introduction

Aneurysmal subarachnoid haemorrhage (aSAH) is widely recognised as one of the most devastating types of stroke and profoundly impacts the quality of life [27, 28]. Despite medical intervention, only a fraction of patients are able to resume their previous lives and work, resulting in significant socio-economic implications. Only 30% of aSAH patients are able to independently manage their daily activities [24, 26]. As a consequence, around 50% of the partners of aSAH survivors work less or not at all after the event. The healthcare costs associated with aSAH are substantial, including hospitalisation, rehabilitation, and the potential long-term loss of young workers [27, 29].

One of the central wishes of patients is survival with the preservation of autonomy. Many report increased fatigue, personality changes, and emotional instability (such as depression or anxiety) even in the absence of neurological deficits, limiting the quality of life (QoL) [9, 15, 24, 32]. Patients find themselves in a situation with new social roles and physical limitations [37].

Numerous conventional predictors, such as age, gender, and neurological status, as well as radiological markers such as the Fisher grade or parameters of perfusion CT imaging [4, 7, 10, 13], are known to predict neurological impairment, typically measured by mRS or GOS, but are not sufficient for determining physical and psychological QoL [37]. This underscores the importance of identifying new indicators for early QoL assessment. To date, few studies have investigated scores and possible predictive factors of QoL [17]. Some studies have proposed long-term prediction of work capacity and health-related QoL using questionnaires and a score for risk stratification of mortality during hospitalisation but do not present predictive models [21, 31].

In 2016, Witsch et al. developed the FRESH score to predict not only the neurological deficits but also the QoL of patients after aSAH. This score allows for a 12-month prognosis of physical outcome expressed as a modified Rankin Scale score (FRESH), cognitive outcome (FRESH-cog), and long-term QoL (FRESH-quol) based on health data available shortly after aSAH [35]. A validation of this score is currently only available for the FRESH score itself, but not for the two scores FRESH-cog and FRESH-quol, which focus more on quality of life.

The present study aimed to validate the FRESH scores in a European aSAH population and compare the newly developed FRESH, FRESH-cog, and FRESH-quol scores with established tools for QoL assessment (SF-36 questionnaire and telephone interview for cognitive status (TICS)) and the real patient long-term outcome in terms of their ability to predict physical disability, cognitive impairment, and QoL after aSAH.

## Materials and methods

All procedures performed in studies involving human participants were in accordance with the ethical standards of the institutional committee, the applicable data protection regulation, and the 1964 Helsinki Declaration and its later amendments. Approval was obtained from the local ethics committee (study ID: 5766R), and written informed consent was obtained for the prospective part of the study. Data will be made available on reasonable request. The manuscript was prepared following the strengthening the reporting of observational studies in epidemiology (STROBE) guidelines [33] and equator network recommendation for preparing scientific manuscripts.

### Inclusion and exclusion criteria

We included all aSAH patients admitted to our tertiary-care hospital between 1st January 2011 and 31st December 2016, who met the following inclusion criteria: (1) received either operative (clipping) or endovascular treatment and (2) were over 18 years old. Additionally, two patients who received primary care externally but were subsequently treated in our hospital were also included. Patients were excluded from the study if they had SAH of non-aneurysmal origin, such as cavernoma or arteriovenous malformation or due to trauma, or if they received only conservative therapy.

### aSAH management

All patients with subarachnoid haemorrhages were treated according to a standardised in-house protocol based on international guidelines [5, 11, 12, 26].

### Data management and definition of outcome measures

The required information for the analysis of aneurysm size, location, morphology, initial symptoms, WFNS and Fisher grading, therapeutic management, and clinical course were collected in digital format from the hospital’s database, including digital documentation programs, physician reports, operative reports, and radiological findings. Radiologic imaging was reviewed using the hospital’s database. The modified Rankin Scale (mRS) was used as a retrospective outcome measure for the degree of disability at 10 to 82 months after haemorrhage.

### Calculation of FRESH scores

The documentation systems “COPRA” and the digital archive “Pegasos” were used as data sources for intensive care data for the calculation of FRESH scores (FRESH, FRESH-cog, and FRESH-quol). These scores were calculated using patient data from the first 48 h after admission, using the app described in the publication and freely available online [35] (https://itunes.apple.com/us/app/fresh-score/id1015675236?mt=8). It should be noted that at the time of the study (April 2017), the app was accessible and, at the time of final manuscript preparation, it was no longer available in the German AppStore. For our study population, the FRESH score was calculated using the following parameters: age (≤ 70 years), Hunt and Hess grade (taken from the WFNS), the use of variables from the Apache II phys score [19, 20], and a “yes/no” answer regarding rebleeding within 48 h. A smartphone application was used for this purpose, as described in the original publication [35]. An iPhone X from Apple, California/USA, with iOS version 11 from Apple, California/USA, and the “FRESH score” app version 2015, Sweta Patel, USA, were used. The formulas of the FRESH scores are depicted in the supplementary information. For FRESH-cog, an inquiry about the so-called “years of education” was conducted by telephone with the study participants. This refers to the total education/teaching/study time (primary school, secondary schools, apprenticeship/training, university). The calculation of FRESH-quol resulted from the previously calculated FRESH and FRESH-cog scores, as well as information regarding the pre-morbid Glasgow Outcome Scale (GOS). The definition of normal values for the calculation of the FRESH scores is given in Supplementary Table 1.

### Short-form 36 (SF-36) questionnaire

Between June and October 2017, 324 information letters containing the short-form-36 (SF-36) questionnaire and an invitation to participate in a telephone interview were sent out [2, 23]. A second mailing was sent to non-responders in August 2017. The SF-36 questionnaire was used as previously published, with its eight subscales (physical functioning, physical role functioning, pain, general health perception, vitality, social functioning, emotional role functioning, mental health) measuring the two main dimensions of physical and mental component scores (PCS and MCS) as a summary scale [23]. In addition, we extended the questionnaire by five questions related to nicotine/alcohol consumption, familial occurrence of aneurysms, presence of hypertension, and work (in)capacity.

### Telephone interview for cognitive status (TICS)

The TICS was conducted over the phone within 7 days of receiving the completed questionnaire or at a scheduled time if the study participant was unavailable. In this context, the current mRS was also assessed via telephone interview. In the event of unanswered questions on the SF-36, a clarification of missing answers was obtained during the TICS. The composition, implementation, and evaluation were conducted as described in previously published literature [1]. The interviews were performed by a single researcher (E.P.G.). Details are depicted in Supplementary Table 2. The time interval between the haemorrhage and the collection of the TICS and the mRS was defined as the individual follow-up time for each patient.

### Statistical analysis and level of significance

IBM SPSS Statistics 25 (IBM, New York, USA, version 25 since August 2017), R (version 3.4.2, GNU Project, Vienna, Austria, since September 2017) and Python 3.9.7 were used for statistical analysis. Numerical variables between two discrete groups were compared using a *t*-test. The relationship between purely numerical variables was analysed using linear regression. The dependency between the dichotomised outcome (mRS 0-2 vs. mRS 3-6) and the FRESH score was modelled using logistic regression. For the age- and gender-adjusted analysis of SF-36 results, they were transformed into PCSz and MCSz, following the comprehensive guidelines provided by Ellert et al. [6], and subsequently analysed. As multiple tests were performed, a significance correction was applied using Šidak’s method to adjust the significance level to *p* = 0.00366 and the trend level to *p* = 0.0075. Only results below these levels were considered significant.

## Results

**Fig. 1:**
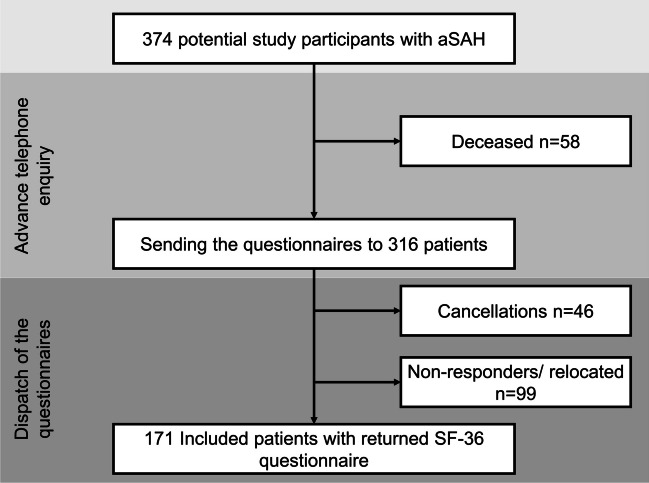
Study population. Depiction of the recruitment process. Non-responders = persons who did not respond to two letters/had relocated to a unknown address. aSAH, aneurysmal subarachnoid haemorhage

The study included 374 participants, out of which 58 had passed away, 46 had who had declined to participate after invitation, and 99 were “non-responders,” meaning they did not respond after two attempts to contact them or had moved and could not be located. Of the remaining patients, 171 (54.1%) responded to the study enquiry; thus, 171 patients were ultimately included in this study. The average follow-up was after 46 ± 24.13 months, with 10 months being the shortest and 82 months being the longest follow-up period. The patient recruitment is shown in Fig. 1, and the patient characteristics are depicted in Table 1.

**Table 1:** Patients characteristics

		*N* = 171
Sex	Female	117 (68.4%)
	Male	54 (31.6%)
Age	Mean ± SD (years)	53.2 ± 11.4
	Minimum (years)	29
	Maximum (years)	79
WFNS	1	79 (46.2%)
	2	19 (11.1%)
	3	24 (14.0%)
	4	22 (12.9%)
	5	27 (15.8%)
Hunt & Hess	0	1 (0.6%)
	1	81 (47.4%)
	2	19 (11.1%)
	3	21 (12.3%)
	4	23 (13.5%)
	5	26 (15.2%)
Fisher	0	3 (1.8%)
	1	10 (6.1%)
	2	23 (13.5%)
	3	45 (26.4%)
	4	89 (52.1%)
Aneurysm location	MCA	36 (21.1%)
	ACOM	65 (38%)
	ICA	1 (0.6%)
	PcaA	8 (4.7%)
	PCOM	37 (21.6%)
	BA	9 (5.3%)
	VA	5 (2.9%)
	PICA	4 (2.3%)
	SCA	2 (1.2%)
	Other	4 (2.3%)
Aneurysm number	More than one aneurysm	35 (20.5%)
Treatment	Surgical	95 (55.6%)
	Endovascular	76 (44.4%)
mRS 10–82 months	0	59 (34.3 %)
	1	50 (29.5 %)
	2	44 (25.7 %)
	3	13 (7.6%)
	4	5 (2.9%)
	5	0
	6	0
Follow-up duration	Average ± SD (months)	46 ± 24.13
	Minimum (months)	10
	Maximum (months)	82

*Acom* anterior communicating artery, *AICA* anterior inferior cerebellar artery, *BA* basilar artery, *ICA* internal carotid artery, *MCA* middle cerebral artery, *mRS* modified Rankin Scale, *N* number of patients, *PcaA* pericallosal artery, *PCOM* posterior communicating artery, *PICA* posterior inferior cerebellar artery, *SCA* superior cerebellar artery, *SD* standard deviation, *VA* vertebral artery

### Patient treatment and complications

Of those included, 46.2% (*n* = 79) received an external ventricular drain (EVD), with 31.8% (*n* = 54) receiving this before the aneurysm treatment. The endovascular approach was preferred in 44.4% (*n* = 76) of patients, while surgical intervention was used in 55.6% (*n* = 95). Manifest intervention-requiring vasospasms occurred in 37.4% (*n* = 64) of patients, and post-haemorrhagic hydrocephalus was detected in 21.1% (*n* = 36) during the initial inpatient stay, while 7 patients showed later secondary hydrocephalus.

A ventriculoperitoneal shunt (VPS) was necessary in 25.1% (*n* = 43) of cases. An infection during hospitalisation was detected in 27% (*n* = 46) of patients, with five cases of dual infections. Pneumonia was diagnosed in 14.6% (*n* = 25) of cases, while 3.5% (*n* = 6) had a urinary tract infection, 2.9% (*n* = 5) had meningitis, 4.7% (*n* = 8) had ventriculitis, and 4.1% (*n* = 7) had wound healing disturbances.

Secondary complications (occurring at least 2 months after the event) occurred in 39.8% (*n* = 68) of cases. These included the development of a secondary hydrocephalus or shunt dysfunction in 14.7% (*n* = 10), cognitive changes (headache, concentration/memory impairment, increased fatigue, reduced drive, dizziness) in 52.9% (*n* = 36), physical impairment (hemisymptomatology, oculomotor paresis, trismus) in 19.1% (*n* = 13), complications in the surgical area (wound healing disturbance/dehiscence, avascular necrosis, hematoma) in 7.3% (*n* = 5), infection (intracranial abscess) in 1.5% (*n* = 1), and newly developed epilepsy in 4.5% (*n* = 3).

### FRESH scores

The distribution of the calculated FRESH scores was as follows: score 1 at 31.6% (*n* = 54), score 2 at 24.0% (*n* = 41), score 3 at 14.0% (*n* = 24), score 4 at 8.2% (*n* = 14), score 5 at 4.1% (*n* = 7), score 6 at 15.8% (*n* = 27), score 7 at 1.2% (*n* = 2), and score 8 at 1.2% (*n* = 2). The average FRESH score was 2.89 ± 1.92, the FRESH-cog was − 5.26 ± 3.91, and the FRESH-quol was − 1.43 ± 2.19.

### SF-36 questionnaire

After evaluating the questionnaire according to the manual, the results of the study population could be projected onto the 8 subscales, as depicted in Table 2. The PCS was on average 46.1, and the MCS was 42.2. The PCS was above average at 21.7% and below average at 31.9% compared to the German normal population of 1994 [23]. The MCS scale was above average at 5.8% and below average at 44.9% compared to the German norm population of 1994 [23].

**Table 2:** Subscales of the SF-36 with corresponding mean values of the study population 10–82 months after bleeding

Subscale	Mean in study population (*n* = 171)
Physical functioning	68.0
Physical role functioning	55.4
Pain	72.1
General health perception	62.2
Vitality	49.9
Social functioning	75.1
Emotional role functioning	58.1
Mental health	64.3

### TICS

In 96.25% (*n* = 154) of the patients who responded to the SF-36 questionnaire, a TICS was feasible. The total score of the study population was on average 29 points ± 4 SD. In the categories “date” and “address,” the highest scores were achieved on average. In contrast, the category “sentence repetition” performed the worst (average score of 0.39 ± 0.49 with a maximum achievable score of 1). The difference between the total scores of the study population (29) and the norm population in 1988 (35.79) was highly significant (*p* < 10^−10^) [1].

### Predicted quality of life (FRESH score) vs. measured quality of life (mRS; TICS; SF-36)

**Fig. 2:**
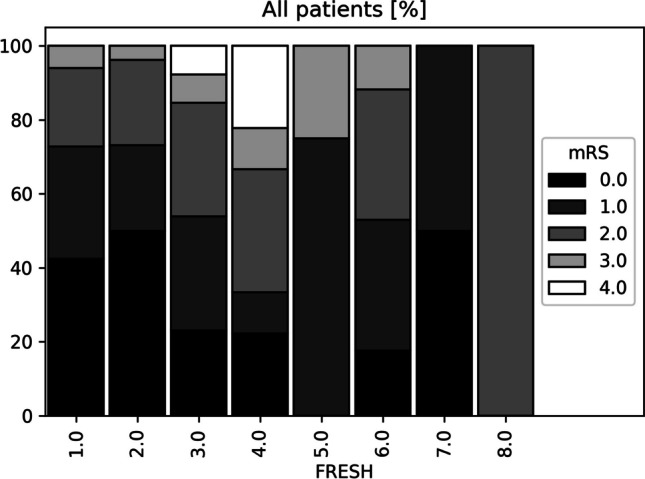
Distribution of the FRESH score in relation to the mRS. Relative distribution of modified Rankin Scale (mRS) score values by respective FRESH score values at 10–82 months (mean ± SD: 46 ± 24.13 months) after subarachnoid haemorrhage

**Fig. 3:**
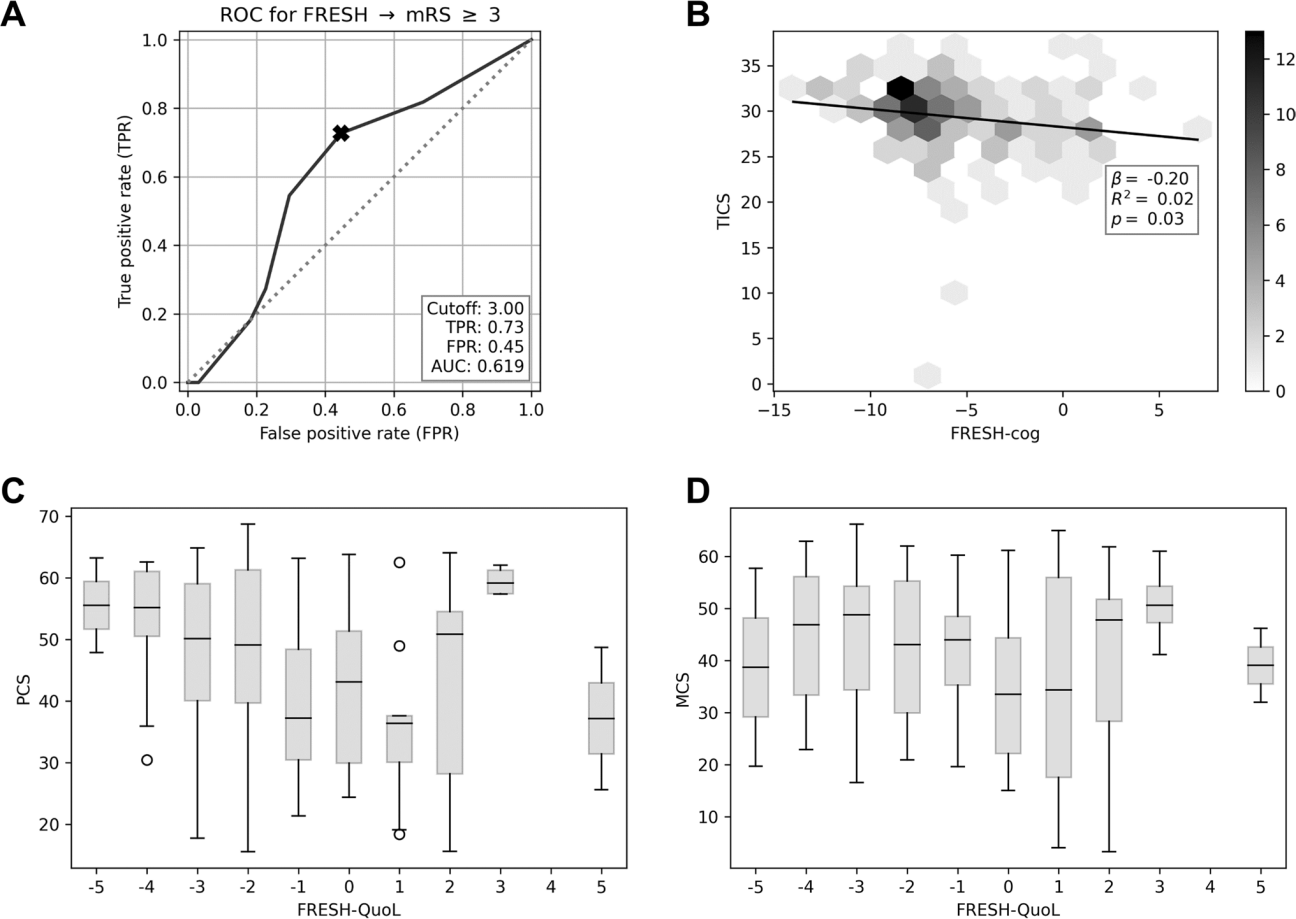
Performance of the FRESH-cog and FRESH-quol scores. **A** Receiver operating characteristic (ROC) curves used for predicting poor outcomes (mRS ≥ 3) using the FRESH score. **B** Depiction of the correlation between FRESH-cog and telephone interview for cognitive status (TICS) scores 10–82 months (mean ± SD: 46 ± 24.13 months) after the haemorrhage. The plot uses a hexagonal binning technique to visualise multiple counts, with shading indicating the number of data points within each bin. The plot legend explains the shading scheme. **C** Correlation between FRESH-quol and the physical component summary (PCS) and **D** the mental component summary (MCS) of the SF-36 questionnaire 10–82 months post bleeding depicted in boxplots as the inter-quartile range and the median as a line

For analysis, the SF-36 was considered in the form of its physical and mental component summary scores. After correction for significance, there was no correlation between the FRESH score and PCS (*p* = 0.09736), MCS (*p* = 0.1796), TICS (*p* = 0.7484), or mRS > 10 months (*p* = 0.024), respectively. The distribution of the mRS scores among the respective FRESH scores is shown in Fig. 2. Even after dichotomisation, there was no correlation between FRESH ≤ 3 and PCS (*p* = 0.0334), MCS (*p* = 0.2064), and GOS (*p* = 0.0406) after correction for significance and the discrimination of the FRESH score between favourable and unfavourable outcome was low (AUC = 0.619; Fig. 3A).

In order to roughly estimate the impact of the time delay between aSAH and follow-up, we built linear regression models including the dichotomized delay (below or above 12 months) and its interaction with the measured quality of life (mRS, TICS, SF-36) as the predictors. *N* = 31 patients had a follow-up under 12 months. No significant relationships were observed between the FRESH and the above quality-of-life measures, the follow-up delay, or its interactions with the FRESH score. Supplementary Table 3 summarises the results.

There was also no significant correlation found for FRESH-cog with TICS after correction for significance (*p* = 0.0311, β = − 0.1985, CI = (− 0.3787, − 0.0183)) (Fig. 3B). Supplementary Table 4 summarises the results for the linear models including interactions with the follow-up delay for the FRESH-cog score. The strongest approximation to significance was found in the analysis “FRESH-quol vs. PCS” with a *p*-value of 0.0204 (β = − 1.1132, CI = (− 2.0525, − 0.1740) (Fig. 3C). When considering “FRESH-quol vs. MCS,” the *p*-value was 0.1361 (β = − 0.7344, CI = (− 1.7025, 0.2336), Fig. 3D), and for “FRESH-quol vs. TICS,” the *p*-value was 0.1608. All of these results were above the defined significance level and did not indicate a significant correlation. Similar results were obtained when accounting for the follow-up delay and its interactions with FRESH-quol (Supplementary table 5).

Additionally, in the analysis of age- and gender-adjusted PCS and MCS of the SF-36 (referred to as PCSz and MCSz), no significant correlations with the FRESH scores were found (PCSz~FRESH= − 7.24, *p* = 0.044, CI = (− 14.27, − 0.19); MCSz~FRESH= − 2.83, *p* = 0.383, CI = (− 9.23, 3.562); PCSz~FRESH-cog= − 3.86, *p* = 0.049, CI = (− 7.89, − 0.03); MCSz~FRESH-cog= − 2.23, *p* = 0.206, CI = (− 5.70, 1.24); PCSz~FRESH-quol= − 6.28, *p* = 0.058, CI = (− 12.77, 0.208); MCS~FRESH-quol= − 2.76, *p* = 0.355, CI = (− 8.64, 3.11)).

When examining individual components of the FRESH scores in this study cohort, there is also no significant correlation with the outcome, as measured by mRS (ordinary least squares regression for correlation with mRS; Hunt and Hess: *p* = 0.128, CI = − 0.076, 0.595; age: *p* = 0.7, CI = − 0.054, 0.036; rebleeding: *p* = 0.809, CI = − 0.927, 1.186).

## Discussion

The key findings of this study can be summarised as follows:

1. No significant correlation could be detected between the actual clinical long-term outcome, as assessed by mRS on average 46 months after aneurysm rupture, and the prognosticated outcome after aSAH as calculated by the FRESH score.
2. No significant correlation was found between the assessed parameters of cognitive performance and the QoL after an average follow-up of 46 months and the FRESH-cog, respectively, the FRESH-quol score.
3. The follow-up delay after aSAH has no significant effect on the estimated outcome and no significant interaction with the FRESH scores.

The core element of this study was a comparison of the well-established and widely validated SF-36 questionnaire, and the TICS with the recently introduced aSAH-specific FRESH scores in terms of predicting quality of life, cognitive status, and physical condition after aSAH [1, 8, 9, 23, 35]. This evaluation is necessary and appropriate and has already been demanded by the authors themselves, since to date, only the FRESH score has been tested based on the mRS, but not the FRESH-cog or FRESH-quol, and the predictive scores were developed with the aim of making ethically justifiable decisions to allocate scarce resources in a more fair way.

In recent years, there have been significant advances in improving the accuracy of prognostic scores for aSAH, leading to an increasing use of these scores in clinical practice [17, 18, 30]. However, validation of these scores is usually difficult, and most scores primarily focus on neurological outcomes (mRS) and do not consider QoL outcomes. This is the main point why the FRESH-quol score holds a special position among the currently available prediction scores [17, 35].

The FRESH score did not show a significant correlation with the mRS between 10 months and 6 years in our study population when adhering to the adjusted significance level. This is surprising as the FRESH score uses the mRS as an outcome scale and should, in theory, predict it accurately. However, it should be noted that the mRS score after 12 months was used to calibrate the FRESH score, initially. Therefore, the FRESH score may not be applicable to the later outcomes represented in our analysis. Yet, in an analysis of patients with a follow-up of less than versus more than 12 months, we observed no significant relationship between the FRESH scores and the quality-of-life measures, the follow-up delay, or its interactions with the FRESH scores. Thus, the longer follow-up compared to the original 12 months does not seem to have a major impact on our analysis. This might be due to the fact that a patient’s health condition tends to change less with increasing time after haemorrhage, at least partially due to aSAH. A possible bias that is more important to consider for a longer observation period like the one partially present in this study is the general cognitive and physical deterioration that comes with age.

Contrary to the results of this study, the primary authors of the FRESH score were able to positively validate the FRESH score in a relatively small patient cohort of 86 patients in 2019 [36]. However, unlike our study, this was again carried out in an American patient population and only included outcome results after 12 months, with a pure validation of the FRESH score based on the mRS [36]. The difference in results may therefore be attributed to the different patient populations, specifically an American for the establishment and the validation of the FRESH scores vs. a European patient population in our study. Extensive evaluations of real QoL, as performed in our study, were not available, and the FRESH-cog and FRESH qual were not validated accordingly. In addition to the small size of the cohort, other limitations of the latter study include the lack of representation of patients with FRESH scores of 7–9 (corresponding to an mRS of 6) due to the study design [36] (also a limitation of the present study). The authors of the score themselves highlighted the limitations of their study, including the fact that FRESH-cog and FRESH-quol could not be externally validated, which remained a problematic point in the follow-up study published in 2019 [36].

Therefore, to the best of our knowledge, our data on QoL are the first to be available for external validation of the FRESH-cog and FRESH-quol. In our European patient cohort, we did not find a significant correlation between the FRESH-cog and FRESH-quol scores, contrary to the initial study in which these scores were established. We used the TICS for the evaluation of FRESH-cog, as in the initial study, resulting in good comparability. However, for the evaluation of FRESH-quol, we used the SF-36, which is an established and widely validated QoL tool subdivided into MCS and PCS [3, 22], unlike the SIP physical score used in the primary study for score establishment. Therefore, the use of a different QoL tool may influence the results. Yet we consider SF-36 as a good readout of real quality of life, as it is an established and commonly used QoL tool across various diseases [2, 23, 34]. With the approach of predicting the QoL by the FRESH-quol, one would expect a significant correlation. However, we did not find a significant correlation between the real quality of life and the calculated FRESH-quol scores in our European study cohort.

The absence of a correlation between the FRESH scores and the outcome, including QoL, in our patient cohort could potentially be a statistical and/or methodological artefact. However, we consider this possibility unlikely. Instead, we believe that the results of this study reflect a true finding. So far, even the individual components of the FRESH scores, such as age or the Hunt and Hess grade, do not correlate with the outcome in our study cohort. A highly plausible explanation for this observed phenomenon, in our view, may be the presence of selection bias, which is a consequence of the retrospective study design whereby only surviving and responding patients were included. Hereby, the patient cohort solely consists of individuals who achieved relatively good outcomes. Consequently, a limited number of patients within this cohort initially presented with high Hunt and Hess or WFNS grades. Therefore, we are predominantly dealing with “mildly” affected patients, for whom age may not significantly influence the outcome. Supporting this notion, previous studies have shown that age has a less prominent role in influencing the outcome in good-grade aSAH patients [16, 25].

Taking this further, it is conceivable that the few patients in our study cohort who initially had poor Hunt and Hess or WFNS grades were possibly misclassified and, in fact, belong to the patients with a rather good outcome. Recent research has demonstrated that the initial WFNS grading, like the Hunt and Hess score heavily relying on the initial vigilance of an aSAH patient, misclassifies some patients with excellent outcomes as “wrong poor grade” aSAH patients [14]. It is conceivable that these few patients in our study cohort, originally classified with high Hunt and Hess grades, represent those initially misjudged individuals. Despite their initial impaired vigilance, these patients are known to achieve excellent outcomes [14]. Consequently, the lack of correlation between the Hunt and Hess grade and the outcome in this study cohort may be a result of this phenomenon. This issue clearly highlights that studies, not only on quality of life (QoL), where feedback from surviving patients is necessary in a retrospective setting, always lead to a significant selection bias. Such studies can not truly represent the entirety of aSAH patients but rather selectively include patients with favourable outcomes, which should be taken into account when planning future studies, especially if the research question relates to patients with poor outcome.

In summary, our study results largely contrast with the existing data, highlighting the complexity of QoL research. Considering all the results of the FRESH scores compared to the SF-36 and TICS, a prospective data collection method after aSAH for future QoL prediction research appears to be a sensible approach. In European patient collectives, it is therefore advisable to use the FRESH scores with caution for planning therapeutic measures until all uncertainties have been resolved.

### Limitations of this study

The main limitation of this study is its retrospective nature. The response rate for the questionnaire and study participation was only 54.1%, which is relatively low. This can be explained by patients’ deaths during the follow-up period or their inability to participate in the study due to an untraceable change of address or other reasons such as a severely impaired neurological condition. The study design hereby leads to a selection bias, as deceased patients and neurologically poor patients (mRS 6 and 5) were underrepresented (depicted in Fig. 2) because they are not able to respond to the questionnaire. Consequently, the study population lacks representativeness, or in other terms, there was an overrepresentation of healthier patients. Another aspect apart from the study design itself that would support this is the observation that participating in a study like this can be emotionally burdensome. In patients with aSAH for whom the development of anxiety disorders, depression, mental fatigue, post-aSAH syndrome, and post-traumatic stress disorder are described, this could lead to another selection bias in the way that more severely affected SAH patients may tend to decline study participation more frequently.

Moreover, the SF-36 questionnaire is susceptible to distortions, especially in older patients with comorbidities and chronic diseases, which was the case in the present study cohort. Finally, a limiting factor of using questionnaires like SF-36 and TICS is the phenomenon of social desirability bias, which can affect the accuracy of the responses. This bias can be particularly problematic when asking about risk factors, physical and mental well-being, and general knowledge (e.g., naming the current president/chancellor, identifying objects), as was the case in this study. The authors of the SF-36 handbook addressed this problem and noted that the test met the criteria for objectivity.

Another important limitation that arises from the retrospective nature of the study is the heterogeneous follow-up time, ranging up to 6 years. The FRESH scores are calibrated to predict outcomes at 1 year, making it unclear whether the scores are significant for outcomes at a later time. Furthermore, based on the ethics vote and the requirement of written consent, we were not able to analyse data of excluded patients to compare them with the included patients, which could have possibly provided further insights into the extent of the selection bias. Lastly, it should be noted that we used an adjusted significance level in the interpretation of the study results. As a result, analyses that would otherwise have been considered significant became non-significant. This could be an indication that the study size was too small, and the analyses in an even larger cohort could turn out to be significant. Yet, in a post hoc power calculation using linear regression with three predictors (FRESH, FRESH-quol, and FRESH-cog), considering the Sidak-adjusted significance level of 0.003 and requiring the detection of an effect of 0.4 with a power of 0.9, the required sample size was calculated to be 150. With the 171 patients included, we therefore also have a certain safety margin with regard to the calculated required sample size.

## Conclusion

In the present monocentric retrospective study, the novel FRESH scores which were designed to predict the outcome, and especially quality of life, after aSAH were compared to the established outcome and QoL parameters in a large European patient cohort with a mean long-term follow-up of 46 months. The analysis failed to show a significant correlation between the predicted outcome and QoL by the FRESH scores and the actually assessed clinical outcome, and we discussed several possible causes for that observation. These results underscore the challenges in researching QoL data and demonstrate the need for prospective studies to further externally validate the FRESH scores.

## Supplementary information

ESM 1 (DOCX 503 kb)

## Abbreviations

aSAH: Aneurysmal subarachnoid haemorrhage
FRESH: Functional recovery expected after subarachnoid haemorrhage
MCS: Mental component summary
mRS: Modified Rankin Scale
PCS: Physical component summary
TICS: Telephone interview for cognitive status
QoL: Quality of life
