# Supplementary material for: Evaluation of FRESH scores in predicting outcome and quality of life after aneurysmal subarachnoid haemorrhage in a European patient cohort
Source: Acta Neurochir (Wien). 2024 Jan 23;166(1):29. doi: 10.1007/s00701-024-05909-2 (PMC10806023; doi:10.1007/s00701-024-05909-2)
Supplement: Supplementary file 1 — (DOCX 503 kb) [file 701_2024_5909_MOESM1_ESM.docx]

Evaluation of FRESH Scores in predicting outcome and Quality of Life after aneurysmal subarachnoid haemorrhage in an European patient cohort

Björn B. Hofmann, MD^1*^, Evgenia Petrova Gundlach, MD^1^, Igor Fischer, PhD^1^, Sajjad Muhammad, MD, PhD^1^, Rainer Kram, MD^2^, Kerim Beseoglu, MD, PhD^1^, Jan F. Cornelius, MD, PhD^1^

^1^ Department of Neurosurgery, Medical Faculty and University Hospital Düsseldorf, Heinrich-Heine-University Düsseldorf, Germany

^2^ Department of Anesthesiology, Medical Faculty and University Hospital Düsseldorf, Heinrich-Heine-University Düsseldorf, Germany

*Corresponding author:

Dr. Björn B. Hofmann

Department of Neurosurgery, Medical Faculty and University Hospital Düsseldorf, Heinrich-Heine-University Düsseldorf, Germany

Phone: 0049 2118117935

e-mail: [bjoern.hofmann@med.uni-duesseldorf.de](mailto:bjoern.hofmann@med.uni-duesseldorf.de)

**Supplementary Information**

**Table of contents**

Page 2- FRESH Scores

Page 3 - Supplementary Table 1

Page 3- Supplementary Table 2

Page 4- Supplementary Table 3

Page 5- Supplementary Table 4

Page 6- Supplementary Table 5

FRESH Scores

**
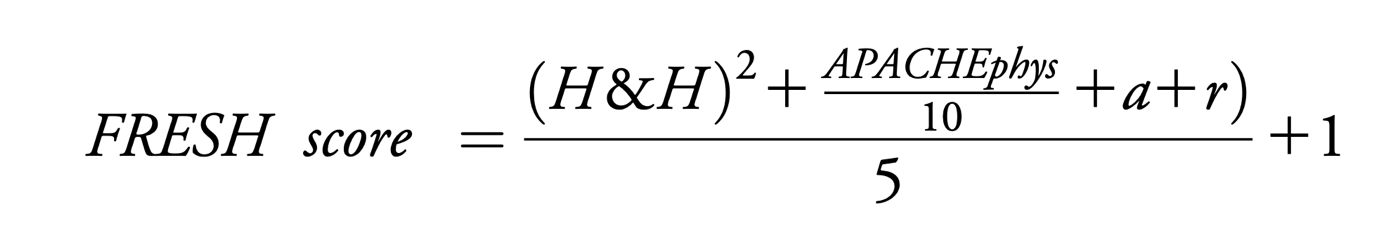
**

**
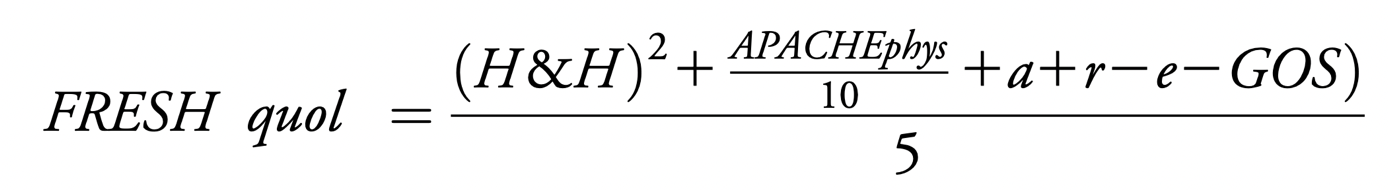
**

**
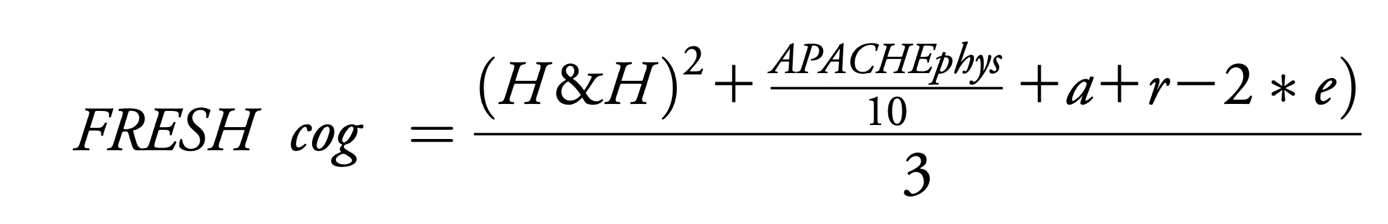
**

| Parameter | Reference Range |
| --- | --- |
| MAP [55] | 70 – 105 mmHg |
| Heart rate (HR) [56] | 50 – 100/min |
| Respiratory rate (RR) [57] | 15 – 20/min |
| Temperature (Temp.) [58] | 36.3 – 37.4 °C |
| White blood cells (WBC) | 4 – 11 x1000/μl |
| Haematocrit (Hct.) | 42 – 50% |
| Sodium (Na) | 135 – 145 mmol/l |
| Potassium (K) | 3.6 – 4.8 mmol/l |
| Creatinine (Cr.) | Up to 1.2 mg/dl |
| PaO2 [40] | 75–97 mmHg or 10–12.9 kPa |
| pCO2 [40] | 35–45 mm Hg or 4.6–6.0 kPa |
| Arterial pH [40] | 7.35–7.45 |

**Supplementary Table 1: Definition of the standard values for the calculation of the FRESH score**

standard values of leukocytes up to Creatinine according to the local Department of Laboratory Medicine. MAP, mean arterial pressure; PaO2, partial arterial oxygen pressure; pCO2, carbon dioxide partial pressure.

| Category | Point Score |
| --- | --- |
| Full name | 1 point for first name, 1 point for last name |
| Date | 1 point for day, month, year, weekday |
| Address | 1 point for house number, street, city, state, zipcode |
| Reverse counting | 2 points if fully correct on first attempt |
| Word list repetition | 1 point for each correct answer Subtractions - max. 3 points |
| Naming specific objects | 1 point per correct answer, max. 4 points |
| Sentence repetition | 1 point for correct repetition |
| Naming chancellor/president with first and last name | 1 point for correct first and last name, max. 2 points |
| Finger tapping on phone | 2 points for audible 5 taps |
| Opposites of words | 1 point per correct answer, max. 2 points |

**Supplementary Table 2: Breakdown of the Telephone Interview for Cognitive Status (TICS)**

|  | β | 95% CI | p |
| --- | --- | --- | --- |
| Outcome variable: PCS |  |  |  |
| FRESH | -3.1710 | (-6.157, -0.185) | 0.038 |
| Follow-up delay | -6.0708 | (-16.732, 4.591) | 0.263 |
| FRESH:delay | 2.6720 | (-0.530, 5.874) | 0.101 |
|  |  |  |  |
| Outcome variable: MCS |  |  |  |
| FRESH | -1.5441 | (-4.606, 1.518) | 0.321 |
| Follow-up delay | 1.1343 | (-9.800, 12.069) | 0.838 |
| FRESH:delay | 0.8575 | (-2.427, 4.142) | 0.607 |
|  |  |  |  |
| Outcome variable: TICS |  |  |  |
| FRESH | 0.0288 | (-0.979, 1.037) | 0.955 |
| Follow-up delay | 0.3686 | (-3.142, 3.879) | 0.836 |
| FRESH:delay | -0.1159 | (-1.200, 0.968) | 0.833 |
|  |  |  |  |
| Outcome variable: mRS |  |  |  |
| FRESH | 0.2639 | (-0.060, 0.588) | 0.109 |
| Follow-up delay | 0.6055 | (-0.479, 1.690) | 0.271 |
| FRESH:delay | -0.1606 | (-0.503, 0.182) | 0.355 |

**Supplementary Table 3 Linear models for predicting the measured outcome by FRESH score, including interaction (:) between follow-up delay and the score.**

|  | β | 95% CI | p |
| --- | --- | --- | --- |
| Outcome variable: PCS |  |  |  |
| FRESH-cog | -1.9468 | (-3.552, -0.342) | 0.018 |
| Follow-up delay | 10.1928 | (-0.817, 21.203) | 0.069 |
| FRESH-cog:delay | 1.4517 | (-0.247, 3.150) | 0.093 |
|  |  |  |  |
| Outcome variable: MCS |  |  |  |
| FRESH-cog | -1.1585 | (-2.820, 0.504) | 0.171 |
| Follow-up delay | 8.8543 | (-2.545, 20.254) | 0.127 |
| FRESH-cog:delay | 0.8968 | (-0.862, 2.656) | 0.316 |
|  |  |  |  |
| Outcome variable: TICS |  |  |  |
| FRESH-cog | -0.3337 | (-0.907, 0.239) | 0.251 |
| Follow-up delay | 1.0614 | (-2.978, 5.101) | 0.604 |
| FRESH-cog:delay | 0.1491 | (-0.455, 0.753) | 0.626 |
|  |  |  |  |
| Outcome variable: mRS |  |  |  |
| FRESH-cog | 0.1287 | (-0.054, 0.311) | 0.164 |
| Follow-up delay | -0.3344 | (-1.636, 0.967) | 0.611 |
| FRESH-cog:delay | -0.0736 | (-0.264, 0.117) | 0.445 |

**Supplementary Table 4: Linear models for predicting the measured outcome by FRESH-cog score, including interaction (:) between follow-up delay and the score.**

|  | β | 95% CI | p |
| --- | --- | --- | --- |
| Outcome variable: PCS |  |  |  |
| FRESH-quol | -3.0580 | (-5.781, -0.335) | 0.028 |
| Follow-up delay | 5.6012 | (-1.592, 12.795) | 0.126 |
| FRESH-quol:delay | 2.1835 | (-0.718, 5.085) | 0.139 |
|  |  |  |  |
| Outcome variable: MCS |  |  |  |
| FRESH-quol | -1.5154 | (-4.327, 1.296) | 0.289 |
| Follow-up delay | 5.2198 | (-2.206, 12.646) | 0.167 |
| FRESH-quol:delay | 0.8401 | (-2.155, 3.835) | 0.580 |
|  |  |  |  |
| Outcome variable: TICS |  |  |  |
| FRESH-quol | -0.3656 | (-1.306 0.575) | 0.443 |
| Follow-up delay | 0.3990 | (-2.160 2.958) | 0.758 |
| FRESH-quol:delay | 0.1511 | (-0.852 1.154) | 0.766 |
|  |  |  |  |
| Outcome variable: mRS |  |  |  |
| FRESH-quol | 0.2727 | (-0.040, 0.586) | 0.087 |
| Follow-up delay | -0.2041 | (-1.034, 0.626) | 0.627 |
| FRESH-quol:delay | -0.1642 | (-0.492, 0.164) | 0.323 |

**Supplementary Table 5 Linear models for predicting the measured outcome by FRESH-quol score, including interaction (:) between follow-up delay and the score.**
